# The Nitrate Transporter (NRT) Gene Family in Poplar

**DOI:** 10.1371/journal.pone.0072126

**Published:** 2013-08-19

**Authors:** Hua Bai, Dejuan Euring, Katharina Volmer, Dennis Janz, Andrea Polle

**Affiliations:** Forstbotanik und Baumphysiologie, Georg-August Universität Göttingen, Göttingen, Germany; Wuhan University, China

## Abstract

Nitrate is an important nutrient required for plant growth. It also acts as a signal regulating plant development. Nitrate is actively taken up and transported by nitrate transporters (NRT), which form a large family with many members and distinct functions. In contrast to *Arabidopsis* and rice there is little information about the NRT family in woody plants such as *Populus*. In this study, a comprehensive analysis of the *Populus NRT* family was performed. Sixty-eight *PtNRT1/PTR*, 6 *PtNRT2*, and 5 *PtNRT3* genes were identified in the *P. trichocarpa* genome. Phylogenetic analysis confirmed that the genes of the NRT family are divided into three clades: NRT1/PTR with four subclades, NRT2, and NRT3. Topological analysis indicated that all members of PtNRT1/PTR and PtNRT2 have 8 to 12 trans-membrane domains, whereas the PtNRT3 proteins have no or up to two trans-membrane domains. Four PtNRT3 members were predicted as secreted proteins. Microarray analyses revealed tissue-specific expression patterns of *PtNRT* genes with distinct clusters of NRTs for roots, for the elongation zone of the apical stem segment and the developing xylem and a further cluster for leaves, bark and wood. A comparison of different poplar species (*P. trichocarpa*, *P. tremula*, *P. euphratica*, *P. fremontii* x *P. angustifolia*, and *P.* x *canescens*) showed that the tissue-specific patterns of the NRT genes varied to some extent with species. Bioinformatic analysis of putative *cis*-regulatory elements in the promoter regions of *PtNRT* family retrieved motifs suggesting the regulation of the NRT genes by N metabolism, by energy and carbon metabolism, and by phytohormones and stress. Multivariate analysis suggested that the combination and abundance of motifs in distinct promoters may lead to tissue-specificity. Our genome wide analysis of the *PtNRT* genes provides a valuable basis for functional analysis towards understanding the role of nitrate transporters for tree growth.

## Introduction

Nitrate (NO_3_^−^) is a major N source for higher plants [1]. A large proportion of the NO_3_^−^ acquired by plants from soil is actively transported through NO_3_^−^ transporters (NRT) [2]. To cope with low (<1 mM) or high (>1 mM) NO_3_^−^ concentrations in soil plant roots have developed high-affinity and low-affinity nitrate uptake systems [3]. Physiological studies revealed that each system is composed of constitutive and inducible components [4]. The plant nitrate transporters form a large family composed of NRT1/PTR (nitrate/peptide transporters), NRT2, and NRT3 members [3]. The members of the NRT family play multifunctional roles in nitrate uptake and transport throughout the plant body [1], [5]. Tissue-specific expression of NRT family genes has been observed in several species, e.g., *Arabidopsis*, rice, and cucumber [5]–[7].

The first nitrate transporter (CHL1 or NRT1.1) was identified in a chlorate resistant mutant of *Arabidopsis* [8]. It takes part in both low-affinity and high-affinity nitrate transport, and also functions as a nitrate sensor to activate the expression of nitrate related genes in plants [9]. Other members of NRT1/PTR are low-affinity nitrate or peptide transporters (PTR). For example, AtNRT1.2 is a constitutive low-affinity nitrate transporter expressed mainly in root hairs and the epidermis of *Arabidopsis* [10]; further three NRT1 members (NRT1.5, NRT1.8 and NRT1.9) are involved in regulating root to shoot long-distance nitrate translocation [1]; and AtNRT1.4 is expressed in petioles and mid-rips with functions for the nitrate allocation to leaves [3], [11].

Peptide transporters (PTR) are also important members of the NRT1/PTR family [12] because they transport di- and tripeptides as well as many other molecules. In *Arabidopsis,* tissue-specific expression was shown for *AtPTR2* in green siliques, roots, and young seedlings. *AtPTR1* and *AtPTR4* are expressed in vascular tissues throughout the plant suggesting a role in long-distance peptide transport [13], [14]. *AtPTR6* is expressed in senescing leaves and pollen [13]. *AtPTR5* is also highly expressed in pollen [15].

Unlike the large subfamily of NRT1/PTR, which consists of 53 members in *Arabidopsis*, only seven NRT2 genes were found in *Arabidopsis*. Single, double, and triple knockout mutants of NRT2.1, NRT2.2, NRT2.4, and NRT2.7 in *Arabidopsis* all have nitrate-related phenotypes and have, therefore, been invoked in nitrate transport [16]–[18]. Most of the AtNRT2 genes are chiefly expressed in the roots, with the exception of AtNRT2.7, whose expression was higher in shoots than in roots [19]. Several recent papers document that the full function of NRT2 proteins is dependent on another family named NRT3 (or NAR2) forming together a two component nitrate uptake system [20]–[23]. The NRT3 family has two members in *Arabidopsis* [24].

Up to now, the molecular features and the regulation of nitrate transporters have been studied mostly in *Arabidopsis thaliana*. With the help of *Arabidopsis* sequences, the whole NRT family has been identified in rice [12] and *Lotus japonicus* [25]. Plett et al. (2010) included eight *NRT1*, five *NRT2*, and four *NRT3* genes of *P. trichocarpa* when comparing *NRT* genes of dicots and monocots [20], but a comprehensive analysis of the NRT family in trees is still lacking. Because poplars are an important feedstock for sustainable bio-energy production [26], the molecular basis of growth stimulation by nitrogen supply is receiving increasing attention [27]. The family of ammonium transporters has been characterized in *Populus* [28]. However, nitrate is the major form of nitrogen uptake for poplars [29] and therefore, more information on poplars nitrate transporters is important. Recently, the expression of a number of genes involved in nitrogen metabolism including two *NRT1*, two *NRT2*, and three *NRT3* genes were compared in two poplar species with contrasting biomass production and revealed distinct patterns in both roots and leaves [30]. However, systematic analyses of the tissue-specific expression patterns of *NRT* genes have not yet been conducted in poplar.

We present here the first complete analysis of the *NRT* family in poplar, including the identification of putative *NRT* genes based on their sequence similarity to *Arabidopsis* genes, their phylogenetic relationships and their expression profiles in different tissues. We furthermore included the prediction of *cis*-regulatory elements (CRE) in the 5′- UTR of all putative *NRT1/PTR*, *NRT2*, and *NRT3* members. CREs play essential roles in regulating gene expression [31], [32]. Previous studies reported several putative NO_3_^−^ response motifs in the *NiR* and *NIA* promoters of *Arabidopsis* [33]–[35]. A 150 bp *cis*-acting element in *AtNRT2.1* promoter was first identified as a NO_3_^–^ specific regulatory region [36]. We used multivariate analysis to identify links between CRE patterns and tissue-specific expression as a first step to understand the regulatory mechanisms of *NRT* genes in poplars. Our results obtained with *P. trichocarpa* and *P. tremula* provide a valuable basis for further studies to gain insights into the functions of the NRTs in poplar.

## Results

### Identification of *NRT* Family Genes

**Figure 1:**
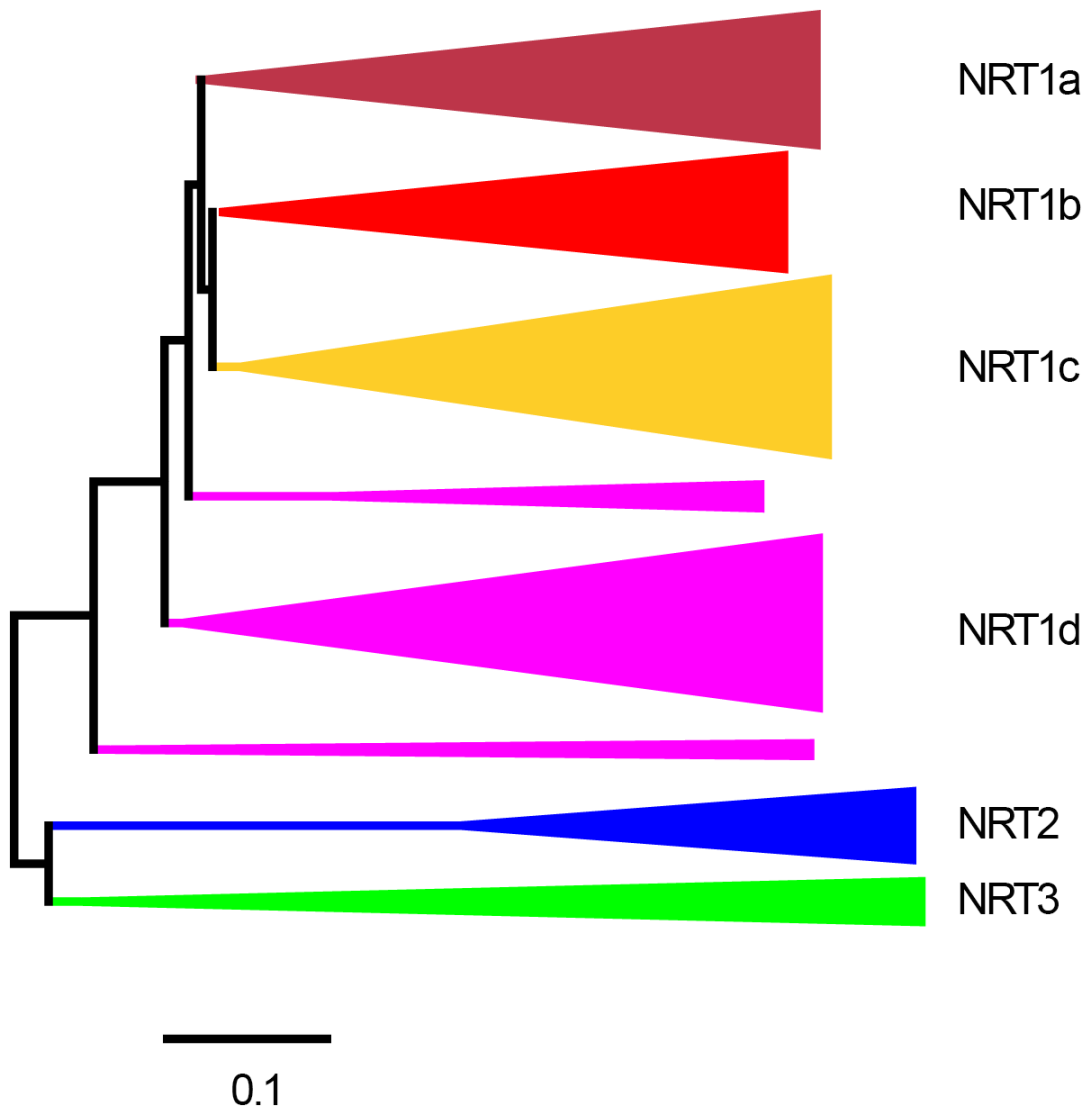
Overview on the phylogeny of the NRT families in *P. trichocarpa* and *A. thaliana*. Detailed information of each clade is presented in figure 2. ClustalX2 was used to perform the alignment of the amino acids and to calculate the final tree with the neighbor-joining method and a bootstrap value of n = 1,000. The tree was displayed using MEGA 5.

**Figure 2:**
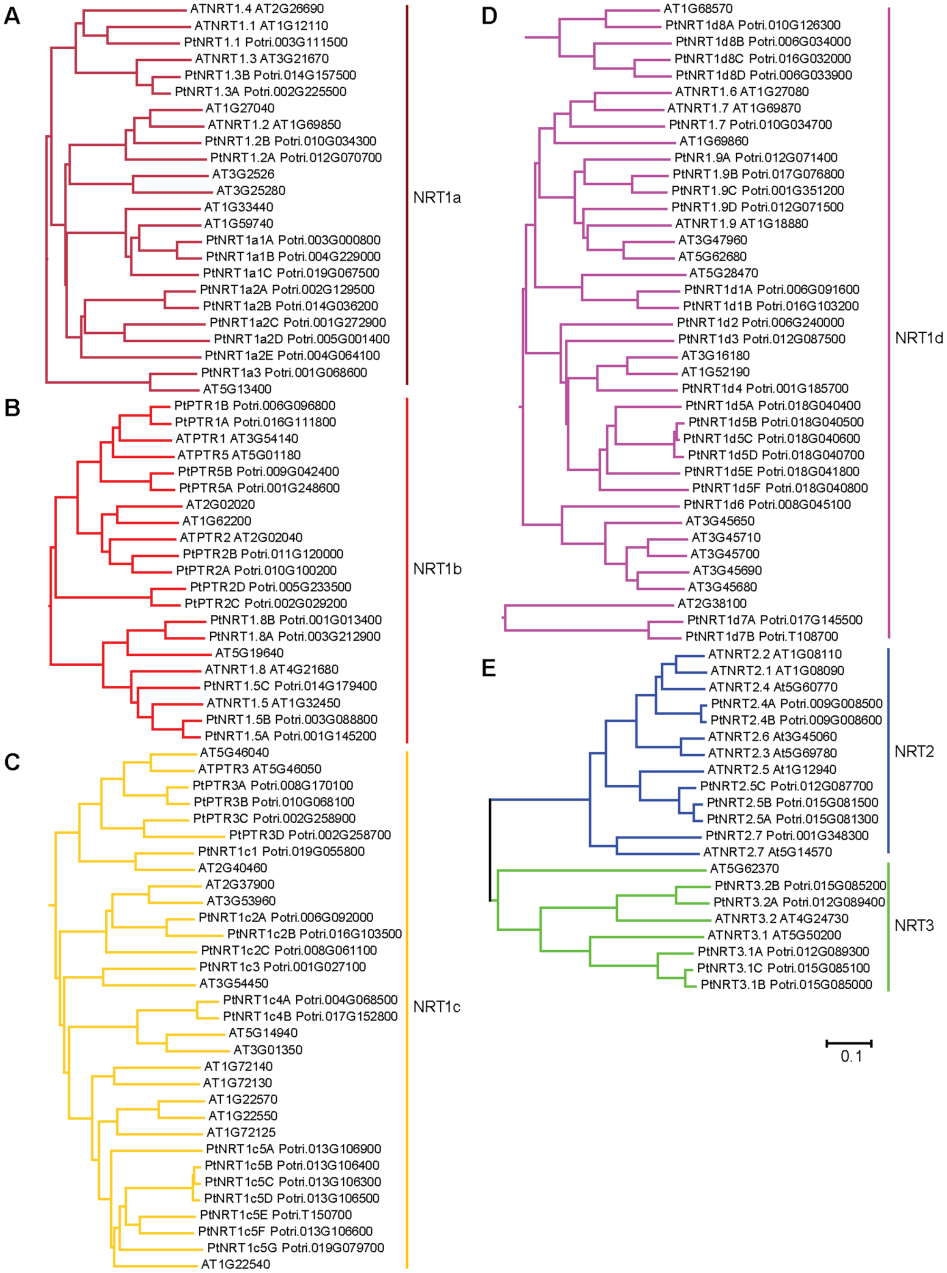
Expanded view of the phylogenetic relationships of the genes in the NRT families of *P. trichocarpa* and *A. thaliana*. **A**. subclade NRT1a. **B**. subclade NRT1b. **C**. subclade NRT1c. **D**. subclade NRT1d. **E**. clade NRT2 and NRT3. **A–D** represent together clade NRT1/PTR. ClustalX2 was used for the alignment of the amino acids and to calculate the final tree with the neighbor-joining method and a bootstrap value of n = 1,000. The tree was displayed using MEGA 5.

To explore the entire NRT family in poplar, we searched the genome of *P. trichocarpa* for *PtNRT* genes based on the full *NRT* family in *Arabidopsis*. We retrieved 68 *PtNRT1/PTR*, 6 *PtNRT2*, and 5 *PtNRT3* genes in poplar (Table S1). We aligned the amino acid sequences of NRT genes of *P. trichocarpa* and *A. thaliana*. The resulting phylogenetic tree defined three main clades with NRT1/PTR, NRT2, and NRT3 (Figure 1). The NRT1/PTR family formed four subclades, which were named PtNRT1a, PtNRT1b, PtNRT1c and PtNRT1d (Figure 2). Consistent with Plett et al. (2010) the names of *NRTs* in poplar were assigned by their sequence similarities with *Arabidopsis NRT* genes [20]. However, as 36 of the 53 *NRT1/PTR* members in *Arabidopsis* have not yet any specific names, we assigned working names to the genes for the purpose of our study (Figure 2, Table S1).

The lengths of the 53 *PtNRT1/PTR* genes range from 1623 bp to 2037 bp with 3 to 6 exons (Table S1). The genes are distributed across 17 of the 19 poplar chromosomes with exception of chromosome 7 and chromosome 15 (Table S1). The lengths of the six *PtNRT2* genes range from 1068 bp to 1593 bp with 2 to 3 exons (Table S1). The *PtNRT2* genes are localized on four chromosomes: *PtNRT2.4A*/*B* on chromosome 9, *PtNRT2.5A*/*B* on chromosome 15, *PtNRT2.5C* on chromosome 12 and *PtNRT2.7* on chromosome 1 (Table S1). The five *PtNRT3* genes have lengths of 618 bp to 1107 bp with 2 to 3 exons and are located on chromosome 12 and chromosome 15 (Table S1).

The known *Arabidopsis* NRT1 proteins are distributed in the subclades PtNRT1a, PtNRT1b, and PtNRT1d (Figure 2). The orthologs of NRT1.1, NRT1.2, NRT1.3, and NRT1.4 in *Arabidopsis* and *Populus* are present in subclade NRT1a (Figure 2A). The orthologs of NRT1.5 and NRT1.8 and peptide transporters (PTR1, PTR2, PTR4, PTR5, and PTR6) in *Arabidopsis* and *Populus* are in subclade NRT1b (Figure 2B). The orthologs of two newly identified *Arabidopsis* glucosinolate transporters (GTR1 and GTR2) [37] together with NRT1.6, NRT1.7, and NRT1.9 in *Arabidopsis* and *Populus* are found in subclade NRT1d (Figure 2D). With the exception of the PTR3 genes, the functions encoded by genes in subclade NRT1c are unknown and names have not yet been assigned to them (Figure 2C). In order to distinguish the unknown poplar NRT1/PTR genes, they were named as PtNRT1+ their subclade assignment and then consecutively numbered (Figure 2, Table S1).

Duplication and multiplication were found in the PtNRT family compared with *Arabidopsis*. We found twelve duplicated gene pairs in *PtNRT1/PTR* present on duplicated chromosomes of *P. trichocarpa* [38] (Figure 2, Table S1). Especially, in subclade NRT1b, 10 out of 13 *Populus* genes are belonging to those paired *PtNRT* genes. We found some multiplicated *PtNRT* genes in subclade PtNRT1a, PtNRT1c, and PtNRT1d. For instance, eight *PtNRT* genes are the orthologs of only two *Arabidopsis AtNRT1s* (At1G33440 and At1G97940) in subclade PtNRT1a. Six *Populus PtNRT* genes are orthologs of At1G22540 in subclade PtNRT1c. In subclade PtNRT1d, six *Populus PtNRT1* genes are similar to At1G52190 and At3G16180. No multiplication was found in clades NRT2 and NRT3. Members of *AtNRT3* are duplicated or triplicated in *Populus*. By contrast, some branches of the phylogenetic tree in clade NRT1/PTR and NRT2 contained more *Arabidopsis* genes than *Populus* genes. For instance, *AtNRT1.1* and *AtNRT1.4* are orthologs of one *Populus* gene named *PtNRT1.1* (Figure 2A). Both *AtNRT1.6* and *AtNRT1.7* are orthologs of *PtNRT1.7* (Figure 2D). Another clear deletion event was found in clade NRT2. One branch of clade NRT2 has two *Populus* genes (*PtNRT2.4A/B*) but five *Arabidopsis* genes including *AtNRT2.1*, *AtNRT2.2*, *AtNRT2.3*, *AtNRT2.4*, and *AtNRT2.6* (Figure 2E).

In order to investigate further properties of the PtNRT proteins, we predicted the hydrophobicity by the TMHMM program (http://www.cbs.dtu.dk/services/TMHMM/). The analysis suggests that all members of PtNRT1/PTR and PtNRT2 have 8 to 12 trans-membrane domains (TMDs) (Table S1). In contrast, PtNRT3.1A/B/C were predicted to have only one to two TMDs, and no TMDs were found in PtNRT3.2A/B. The prediction of signal peptides showed that 12 NRT genes encoded putatively secreted proteins (Table S1). Four of those are belonging to the PtNRT3 (PtNRT3.1A/B/C and PtNRT3.2A) family. The other eight putatively secreted proteins are belonging to the PtNRT1/PTR cluster (Table S1). The prediction of signal peptides was also performed for *Arabidopsis*. We found that AtNRT3.1 and At2g38100, which is the closest ortholog to *PtNRT1d7A,* are also putatively secreted proteins (Table S1). Overall, these analyses reveal that the structures of the PtNRT3 proteins are distinctly different from those of the PtNRT1/PTR and PtNRT2 genes.

### Differential Expression of *NRT* Genes in Poplar

In order to explore the expression patterns of poplar *NRT* genes we compiled microarrays from public databases and own analyses for six tissues including the elongation segment of the stem top (T), the developing xylem (DX), wood (W), bark (B), leaves (L), and roots (R) (Table S2). The data base encompassed five poplar species. Significant signal intensities were detected for 57 *PtNRT* genes on the microarrays (Table S1). Eleven genes have no probesets and eleven genes were not expressed on the microarrays (Table S1). For three of the latter group, quantitative real time polymerase chain reaction (qRT-PCR) data are available (see below under “tissue-specific expression”) suggesting that the expression of these silent genes was too low for detection by microarray analyses. This may also be true for the remaining eight genes or they may be expressed only under specific conditions.

**Figure 3:**
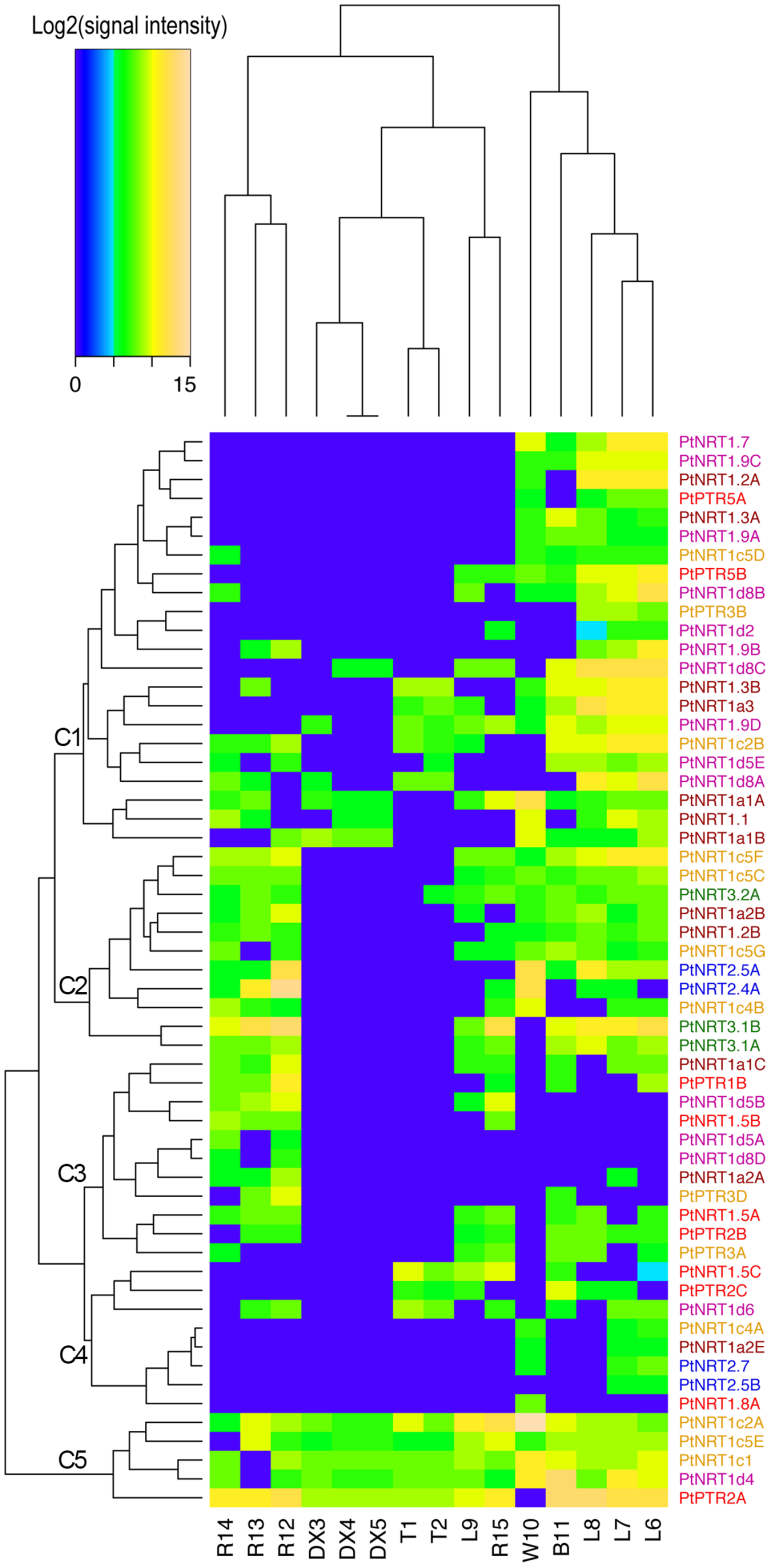
Expression profiles of *NRT* family genes in *Populus* across different tissues. The heatmap represents the hierarchical clustering of average log_2_(signal intensity) of *Populus NRT* genes in various tissues. T, stem top; DX, developing xylem; L, leaves; W, wood; B, bark; R, roots. The numbers refer to different experiments compiled in Table S2. The color of the gene name represents the clade in the phylogenetic tree (Figure 1). The Affymetrix microarray data were obtained from ArrayExpression database (http://www.ebi.ac.uk/arrayexpress) and their accession number are shown in Table S2.

Hierarchical clustering of the available dataset revealed clear differential expression of *PtNRT* genes in different poplar tissues (Figure 3). The tissues were separated into three clusters: The NRT expression patterns in roots from *P.* x *canescens*, *P. euphratica* and *P. tremula* formed one cluster, that of the developing xylem and tip together with roots and leaves of axenically grown *P. trichocarpa* formed another cluster and the third cluster encompassed leaves of *P. fremontii* x *angustifolia* and wood, and bark of *P. trichocarpa*. Based on the expression patterns in the six tissues analyzed, the *PtNRT* genes were grouped into C1 to C5 (Figure 3). All 22 genes in C1 were expressed in leaves, and 12 of those were also expressed in bark and wood. Genes in C2 were expressed in the roots, leaves, bark, and wood, but not in the developing xylem and tip. All genes in C3 were expressed in roots, and some of those had expression in other tissues. Genes in C5 were expressed in all six tissues. We found that most of the *PtNRT* genes were expressed in leaves, bark, and wood, whereas a lower number of *PtNRT* genes were expressed in roots (Figure 3). Very few genes were found in the developing xylem and the elongation zone, all belonging to the *PtNRT1/PTR* group (Figure 3).

### Comparing the Tissue-specific Expression of NRT Genes in *P. tremula*

We selected 21 poplar *NRT* genes (13 *NRT1*, 5 *NRT2*, and 3 *NRT3*), whose othologs had previously been functionally characterized in *Arabidopsis* [1] for qRT-PCR in *P. tremula*. Eight tissues of *P. tremula* were used including fine roots (FR), coarse roots (CR), developing xylem (DX), wood (W), bark (B), stem top (T), leaves (L), and petioles (P).

**Figure 4:**
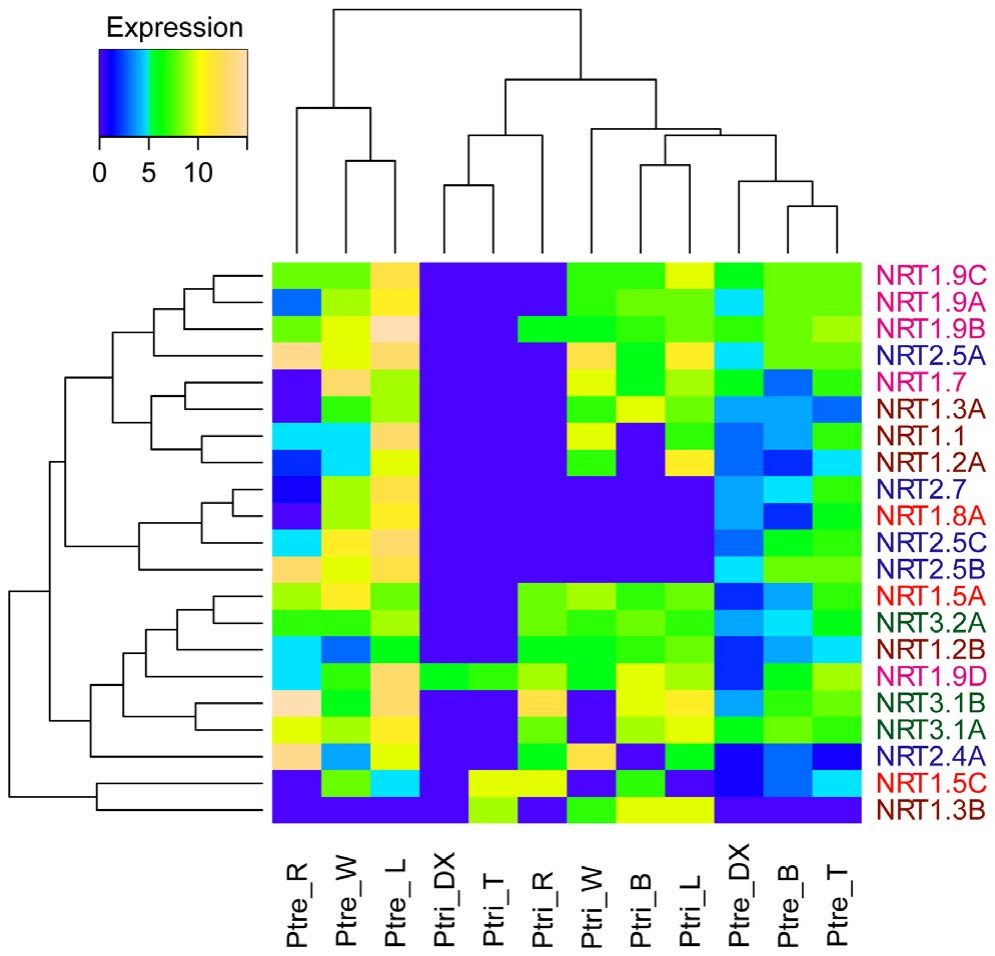
Comparison of tissue-specific expression patterns of *NRT* genes in *P. trichocarpa* (Ptri) and *P. tremula* (Ptre). The heatmap represents the hierarchical clustering of average log_2_(relative expression) of *NRT* genes in stem top (T), bark (B), developing xylem (DX), leaves (L), wood (W), and roots (R). The color of the gene name represents the clade in the phylogenetic tree (Figure 1).

A heatmap was generated to compare the expression of *NRT* genes of *P. tremula* (PtreNRT) with those of *P. trichocarpa* (PtNRT). Hierarchical clustering revealed a clear separation of the two poplar species (Figure 4). Higher signal intensities were observed in *P. tremula* analyzed by qRT-PCR than in *P. trichocarpa* analyzed by microarrays. Tissue-specific expression of the selected *PtreNRT* genes was observed (Figure 4). Leaves, wood, coarse roots, and petioles formed one cluster. Most of the tested *PtreNRT* genes were highly expressed in those tissues. Developing xylem, bark, and stem tip formed another cluster, which contained fewer expressed *PtreNRT* genes (Figure 4). We found that bark exhibited diverging expression of *NRT* genes in two poplar species (Figure 4). In *P. trichocarpa NRT* genes in bark clustered with leaves, but in *P. tremula* with developing xylem and stem top. In summary, the results of the qRT-PCR confirmed a clear separation of *NRT* gene expression in different tissues and pointed to species related differences.

### *Cis* - regulatory Elements of Nitrate Transporters in *P. trichocarpa*

To investigate whether the differential regulation of the *P. trichocarpa NRT* genes might be related to differences in regulatory motifs, the 1 kb UTRs of all *PtNRT1*, *PtNRT2*, and *PtNRT3* genes were analyzed using the PLACE signal scan program. This analysis resulted in 142 different motifs, many of which are related to development, stress, or phytohormones (Table S3). A cluster analysis divided the predicted CREs into 14 groups named as MC1 to MC14 (Figure S1). MC1 contained 9 motifs present in the promoter regions of all *NRT* genes, namely CAATBOX1, ARR1AT, WRKY71OS, ROOTMOTIFTAPOX1, CACTFTPPCA1, DOFCOREZM, GATABOX, GT1CONSENSUS, and GTGANTG10 (Table S3). These motifs were also the most abundant motifs with more than 11 copies per gene and per motif.

The cluster analysis furthermore showed that the *PtNRT* genes were divided into nine groups from GC1 to GC9, based on the enrichment of CREs (Figure S1). We found that *PtNRT2* genes were all clustered in GC6, and the *PtNRT3* genes in GC8. The *PtNRT1/PTR* clustered in 8 different groups based on their motif patterns. This analysis highlights clear differences of the CRE patterns in the promoters of the *PtNRT1/PTR*, *PtNRT2*, and *PtNRT3* families, respectively.

**Figure 5:**
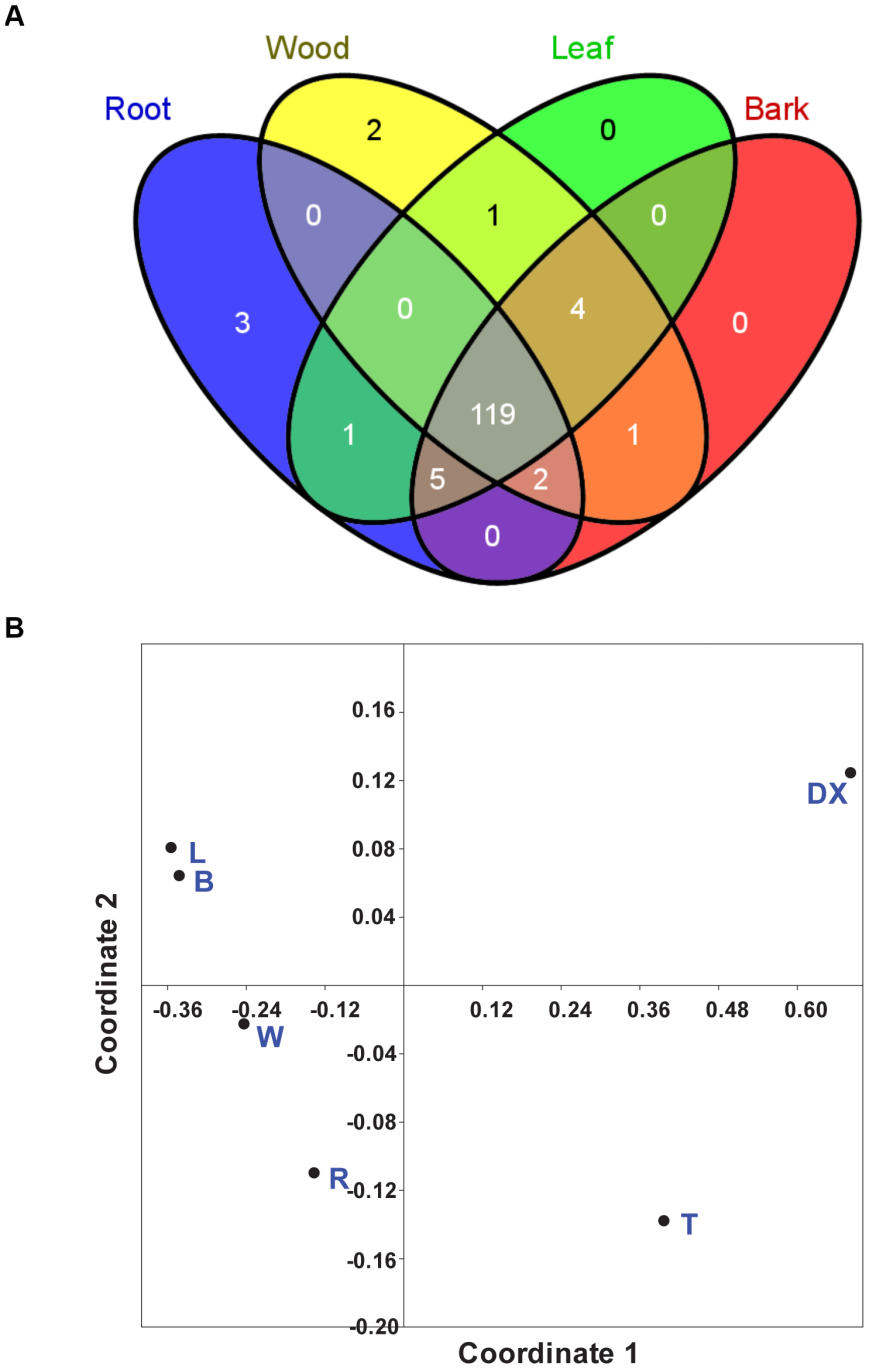
*cis*-regulatory elements of *NRT* genes in *P. trichocarpa*. **A.** Overlapping CREs of *NRT* genes in leaves, bark, wood, and roots. The diagram shows the number of CREs in *PtNRT* genes expressed in specific tissues. The Venn diagram was drawn using VENNY (http://bioinfogp.cnb.csic.es/tools/venny/). **B.** Nonmetric multidimensional scaling (NMDS) ordination of CREs abundance and tissue specific expression of *PtNRT* genes. T, stem top; DX, developing xylem; L, leaves; W, wood; B, bark; R, roots. The data matrix used for the NMDS analyses is shown in Table S3.

In order to explore potential relationships between the motif patterns and the tissue-specific expression of *PtNRT* genes, we compared the presence and absence of known motifs across the analyzed tissues (Figure 5A). Most of the motifs (119/136 in *P. trichocarpa*) were present in the four main tissues leaves, bark, wood and roots (Figure 5A). Roots contained three (ACGTTBOX, ACGTOSGLUB1, E2F1OSPCNA) and wood contained two unique motifs (LRENPCABE, ABREATCONSENSUS), whereas no unique motifs occurred in leaves, bark, developing xylem or tip. Therefore, tissue-specificity of the observed NRT expression patterns cannot be related to the presence or absence of known CREs. Multivariate analysis of motif abundance in the different tissues identified similar patterns for bark and leaves, and distinct patterns for all other tissues (Figure 5B). These results suggest that different regulatory mechanisms may act in specific tissue controlling *PtNRT* gene expression.

## Discussion

### The Poplar Genes Families NRT1/PTR and NRT3 but not NRT2 are Expanded Compared with *Arabidopsis*

Here, we present a comprehensive analysis of *NRT* related genes in poplar. We retrieved a total of 79 sequences with high similarities to the 62 known *Arabidopsis* genes. In a preceding analysis Plett et al. (2010) included 8 *PtNRT1*, 5 *PtNRT2* and 4 *PtNRT3* genes for *in silico* analysis of *NRT* genes of dicots and monocots [20]. The present identification of *PtNRT* family was based on newly characterized *NRT* genes of *Arabidopsis* (*AtNRT1.9*, *AtGTP1*, and *AtGTP2*) in addition to those already available before 2010 [37], [39] and relied on an improved annotation of the poplar genome (*Populus trichocarpa* v3, DOE-JGI, http://www.phytozome.net/poplar). Similar to other species like *Arabidopsis*, rice and *Lotus*, a high number of *NRT1/PTR* genes and a comparatively low number of *NRT2* and *NRT3* genes were retrieved. In comparison with *NRT1/PTR* in *Arabidopsis* (53 genes) and *Lotus* (37 genes), in both rice (80 genes) and poplar (65 genes) the *NRT1/PTR* family was expanded [12], [25].

The *P. trichocarpa* genome is duplicated [38] and therefore, contains more protein-coding genes than *Arabidopsis*, ranging on average from 1.4 to 1.6 putative *Populus* genes for each *Arabidopsis* gene [38]. Whole-genome duplication raises the rate of gene gains and losses [40]. Many studies presented evidence for gene duplication in *P. trichocarpa* such as the analysis of the complete glutamine synthetase family [41]. A high proportion of the members of the oligopeptide transporter family in *P. trichocarpa* might be derived from tandem duplication [42]. Our phylogeny based on full-length NRT amino acid sequences revealed that gene expansion and loss occurred also in the *NRT* family of *P. trichocarpa* compared with *Arabidopsis*. We found thirteen duplicated *PtNRT1/PTR* two NRT3 gene pairs in *P. trichocarpa* compared with *Arabidopsis*. For example, three closely related orthologs of *Arabidopsis AtNRT2.5* were identified in *Populus*. Three branches in clade NRT1/PTR had one to two *Arabidopsis AtNRT* genes but seven to eight *Populus PtNRT* genes. These results suggest that duplications and multiplications have contributed to the expansion of the *PtNRT* gene family in *Populus*.

However, a loss of *NRT* genes was also observed in NRT1/PTR and NRT2 of *P. trichocarpa*. For example, *AtNRT1.1* and *AtNRT1.4* are the orthologs of one poplar gene (*PtNRT1.1*). *AtNRT2.1*, *AtNRT2.2*, and *AtNRT2.4* in *Arabidopsis* have complementary functions in high affinity nitrate transport [43]. The phylogeny showed that AtNRT2.1, AtNRT2.2, AtNRT2.4, together with AtNRT2.3 and AtNRT2.6 are the orthologs of only two poplar proteins (PtNRT2.4A/B). The functional significance of these results will have to be addressed in future studies. Our current analysis provides a valuable basis for a systematic approach to evaluate nitrate uptake and translocation in poplar.

In *Arabidopsis*, topology predicted that both NRT1s and NRT2s contain 12 putative TMDs with a large hydrophilic loop between TM6 and TM7 [12]. Similarly, we found that the predicted poplar NRT1 and NRT2 proteins have 8 to 12 TMS. In contrast to the NRT1 and NRT2 members of *Arabidopsis* and *P. trichocarpa*, the NRT3 proteins in *Arabidopsis* and *P. trichocarpa* have either no or few (up to two) TMDs. In addition, PtNRT3.1A/B/C, PtNRT3.2A, and AtNRT3.1A were predicted as secreted proteins. Currently, experimental evidence for NRT3s as secreted protein is missing. However, NRT3 proteins are soluble proteins that have been shown to form a two component complex with NRT2 proteins for high affinity nitrate uptake [5], [21]–[23], [44].

RT-PCR analysis in *Arabidopsis* revealed that 51 of the 53 *NRT1/PTR* genes are expressed, whereas two genes could be pseudogenes [12]. To examine whether the identified *PtNRT* genes in poplar are expressed, we studied the transcript abundance of 79 *PtNRT* genes using microarray experiments representing six tissues in *Populus*. The results indicate that 57 *PtNRT* genes were expressed, 11 *PtNRT* genes exhibited no signal, and 11 *PtNRT* genes had no probesets on the arrays. However, 8 of the silent *PtNRT* genes in *P. trichocarpa* were expressed in other poplar species, which indicates that some *PtNRT* genes might have species-specific expression in *Populus* or may be pseudogenes For example, *PtNRT3.2B* was silent on the microarrays and we have not been able to amplify this gene by qRT-PCR. Therefore, *PtNRT3.2B* could be a pseudo gene. Among the selected *PtNRT* genes, *PtNRT1.8B, PtNRT2.4B, PtNRT2.5C,* and *PtNRT3.1C* do not have probesets on the microarrays. One possible reason might be their high sequence similarities with other family members. Our PCR amplification of *PtNRT1.8B* and *PtNRT2.5C* showed positive results, and suggested that *PtNRT1.8B* and *PtNRT2.5C* are expressed genes. In addition, a study in *P. popularis* and *P. alba × P. glandulosa* showed the expression of *PtNRT2.4B* and *PtNRT3.1C* [30]. Therefore, all members of *PtNRT2* and *PtNRT3* except *PtNRT3.1B* are expressed genes. Eighteen *PtNRT1/PTR* were not detected as expressed genes. They could be pseudogenes or expressed in other conditions.

### Tissue-specific Expression of *NRT* Genes in Poplar

The members of the NRT1/PTR, NRT2, and NRT3 family play different roles in nitrate uptake and transport throughout the plant body [1], [5]. Our data show that tissue specific expression of *NRT* genes as documented for *Arabidopsis* and rice [5]–[7] is also true for poplar. The distinct expression patterns of the *PtNRT1/PTR*, *PtNRT2*, and *PtNRT3* genes suggest diverse biological functions in specific tissues. The tip and the developing xylem contained a much lower number of expressed *NRTs* than the other tissues and the expressed *NRTs* were subsets of wood, leaf, and bark. Unique tissue-specific *NRTs* were *PtNRT1.2A, PtNRT2.7, PtNRT1a2E, and PtNRT1c4A* in leaves and wood, *PtPTR3B* and *PtNRT2.5B* in leaves, *PtNRT1.8A* in wood, and *PtNRT1.5B*, *PtNRT1d5A* and *PtNRT1d5B* in roots. Three of these genes have *Arabidopsis* orthologs with known functions: AtNRT1.2 is a low affinity nitrate transporter that has recently been shown to be also an abscisic acid transporter [45]. AtNRT2.5 is a high affinity nitrate transporter important for growth regulation, which is mainly expressed in leaves of *Arabidopsis* [46], and AtNRT1.5 is involved in xylem loading of nitrate in roots [47]. NRT1.8 has functions in nitrate removal from the xylem sap in *Arabidopsis* [21], and we found that PtNRT1.8A was specifically expressed in wood of *P. trichocarpa*.

The nitrate transporter/receptor *AtNRT1.1* ( =  nitrate tranceptor *CHL1)* was the first identified NRT in *Arabidopsis* and is currently the most extensively studied gene in this family [48]. *AtNRT1.1* is expressed widely in roots and shoots with highest expression in the epidermis of the tips of the primary roots; it is also expressed in the endodermis of older roots and weakly expressed in mature parts of roots [49]. It has multiple functions as a nitrate transporter and sensor in roots [9]. It is furthermore expressed in guard cells and functions in stomatal opening [50]. It can also transport auxin, which may explain its participation in the regulation of lateral root growth [51], [52]. In *P. trichocarpa* its expression was found in leaves and wood, but not yet in roots. This finding was unexpected because a sensor is anticipated to be constitutively present, particularly in roots the primary uptake sites for nitrate. However, if the sensor is relatively stable, its expression can be low. It is also possible that *NRT1.1* when expressed in the root tip, where the major NO_3_^−^ flux occurs [53] was diluted in extracts of the whole poplar root system and therefore not detected. Indeed, Li et al. (2012) reported low *NRT1.1* expression in roots of two different poplar species, which increased in response to N fertilization [30]. Notably roots contained the highly expressed ortholog *PtNRT1.2B* to the low affinity nitrate/abscisic acid transporter of *Arabidopsis*, while leaves contained homolog of this gene (*PtNRT1.2A*). These findings may point to links between the regulation of nitrate uptake and drought stress, which are also corroborated by the CRE analysis (see below).

To transport NO_3_^−^ between roots and the aerial parts, NO_3_^−^ has to be loaded to the vascular tissues [1]. Many xylem and phloem located *NRT* genes are belonging to the *NRT1/PTR* family [1], [54]. For instance, in *Arabidopsis,* AtNRT1.5, AtNRT1.7, AtNRT1.8 and AtNRT1.9 are expressed in transport tissues and function in long-distance transport or remobilization of NO_3_^−^ [1], [54]. In line with these studies, we identified the expression of their poplar orthologs in roots, bark, wood, and leaves. In *Arabidopsis,* the members of the AtNRT1/PTR family transport not only nitrate but also auxin, carboxylates, di- and tripeptides, and even glucosinolates, and it is assumed that other substrates might be identified in the future [12], [13], [37]. For poplar, we still have no experimental evidence for their natural substrates.

In contrast to the *NRT1/PTR* family, none of the *PtNRT2* and *PtNRT3*–related transcripts was detected in the developing xylem and the stem tip by microarray analysis. In *Arabidopsis*, rice, and peach the expression levels of *NRT2* genes are generally higher in roots than in shoots [5], [7], [24], [55], [56]. One exception is *AtNRT2.7* that exhibits a strong leaf-specific expression and participates in balancing the leaf nitrate content [57]. This notion may also be true for poplar, where *PtNRT2.7* exhibited high expression levels in leaves.

In *Arabidopsis*, AtNRT2.4 participates in phloem nitrate transport [16]. In poplar bark, we did not detect the expression of *PtNRT2.4A*, but that of *PtNRT2.5A*. The finding that *PtNRT2.5A* clustered together with *PtNRT3.1A/B* may suggest that these proteins are required together for high affinity transporter of nitrate in the bark. Poplar *PtNRT2.4* was abundant in roots, wood including the stem tip, and leaves, pointing to participation in long distance transport from the belowground to the above ground tissues.

Interestingly, with the exception of *PtNRT1.9D*, which is an ortholog to an *Arabidopsis* nitrate transporters in the phloem [39], none of the *NRT* genes with similarity to known *Arabidopsis* nitrate transporters was present in all analyzed *P. trichocarpa* tissues. *NRT1/PTR* genes constitutively expressed in all tissues were members of subclade NRT1c and subclade NRT1d, whose *Arabidopsis* orthologs were classified as members of the major facilitator superfamily involved in oligopeptide transport. However, distinct functions of these NRT1 members are still unclear. A further constitutive *PtNRT1c1* (subclade NRT1c) has an *Arabidopsis* ortholog which can transport di- and tripeptides but not NO_3_^−^ in oocytes assays [58]. Therefore, most genes found across all *P. trichocarpa* tissues do not appear to be specific for NO_3_^−^ transport.

### *Cis-*regulatory Elements Related to Specific Expression of *PtNRTs* in Tissues

CREs play essential roles in regulating gene expression pattern [32]. Here, nine of a total of 142 CRE motifs were found in the promoter regions of all *PtNRT* genes (Cluster MC1). Two of these motifs (GATABOX, DOFCOREZM) are involved in N metabolic pathways [33], [59], [60]. For instance, in spinach, GATA motifs exist in the promoter region of the nitrite reductase gene that is differentially regulated by ammonium [33]. Transgenic *Arabidopsis* plants overexpressing Dof1 showed better growth under low-nitrogen conditions [61]. The CAAT motif of CAATBOX1 in cluster MC1 is a general motif for binding of transcription factor in eucaryotes (identified e.g. in the promoter of the legumin) [62]. The GT1CONSENSUS motif is present in the promoter of many light-regulated genes such as the rubisco small subunit [63]. CACTFTPPCA1 is important for the regulation of phosphoenol pyruvate carboxylase [64], a central enzyme for anapleurotic reactions, which are required when metabolites are withdrawn from the citrate cycle for amino acid biosynthesis, e.g. that of glutamate [65]. The ubiquitous presence of these motifs suggests that the *PtNRT* genes underlie a tight regulation by N as well as plant energy and carbon metabolism. Further motifs in MC1 are related to organ specificity (ROOTMOTIFTAPOX1 [66]) and hormone regulation (ARR1AT, WRKY71OS) [67], [68]. ARR1AT is a binding element [69], [70] for *ARR1*, which is a cytokinin response transcriptional activator [67], [68]. WRKY71OS, a binding element for *WRKY71*, encodes a transcriptional repressor of gibberillic acid signaling [71] and is induced by cold, salt and dehydration stress in *Musa spp.* [72]. Because gibberillic acid and cytokinin are both plant growth hormones our findings place *PtNRT* genes into a growth regulatory net.

Many of the other motifs, which did not occur in all NRT promoters, still may indicate functional redundancy with the common regulatory elements because we detected a large number of motifs for light (12), sugar (8), hormone (16) and nitrogen regulation (14). Among the hormone-related motifs not only growth, but also abscisic acid-related motifs were identified, which points to drought stress regulation of some NRTs. This notion is also supported by a considerable number of motifs (11) for Myb transcription factors also known to play roles in drought stress [73]–[76].

In addition to ROOTMOTIFTAPOX1 we found further CREs related to tissue-specific expression such as NODCON1G/NODCON2G [77], POLLEN1LELAT52 [78], and TAAAGSTKST1 [79]. However, we could not find a direct relationship between the enrichment of these motifs and the tissue-specific expression of their corresponding *PtNRT* genes. A possible reason for this lack of correlation could be that CREs are not working independently of each other. For example, Ye et al. (2012) found two *cis*-regulatory elements (GSE1 and GSE2) important for the expression of genes in green tissues in rice [80]. GSE1 and GSE2 harbored three *cis*-elements, namely ROOTMOTIFTAPOX1, NODCON2GM, and DOFCOREZM, which appeared to be required together for tissue-specific expression. Our analysis of motif occurrence in the analyzed tissues revealed strong overlap (119/138 = 86%) in leaves, bark, wood and roots. Only two motifs were specific to wood and three to roots, and none to the other tissues analyzed. The specific motifs were very rare, each occurring only in one NRT gene and showed no correlation with distinct tissues. This observation precludes a role in tissue-specificity. Our NMDS analysis suggests that the combination and abundance of motifs in distinct promoters may lead to tissue-specificity because most tissues were clearly separated. Only for leaves and bark our analysis predicts similar regulation.

In conclusion, the results of this study display the identification and an expression analysis of the whole *NRT* gene family in *Populus.* The prediction of *cis*-regulatory elements suggests various possible regulatory mechanisms of *PtNRT* genes, in particular with regard to phytohormones. These data provide a valuable basis for further studies to gain insights into the functions of nitrate transporters in poplar.

## Materials and Methods

### Sterile Growth of *P. trichocarpa*

*P. trichocarpa* were multiplied by micropropagation and grown under axenic conditions in with autoclaved sand-filled (Ø 0.71–1.25 mm Melo, Göttingen, Germany) Petri dishes supplied with Woody Plant Medium [81]. The plants were grown for five weeks in an acclimatized room (26°C, 60% relative air humidity, day/night length of 16/8 h with a photosynthetic active radiation (PAR) of 150 µmol photons m^−2^s^−1^). Then the plants were separated into roots and shoots, shock-frozen in liquid nitrogen and stored at −80°C. For transcript profiling, materials of 15 plants per experiment were pooled, ground in a ball mill (Retsch, Haan, Germany) and used for RNA extraction with a modified method after Chang et al. (1993) [82]. Three independent biological samples were obtained from three experiments. Total RNA was purified with the RNeasy mini kit (Qiagen, Valencia, CA). RNA integrity was assessed on an Agilent 2.100 Bioanalyzer (Agilent, Santa Clara, CA) and analysed on the GeneChip® Poplar Genome Array (Affymetrix, Santa Clara, CA) at the Microarray Facility Tübingen. Raw and normalized data are available at the ArrayExpress database under MEXP-3909 and MEXP-3910 (further information: Table S2).

### Plantlets of *P. tremula* for the Transcript Analysis of *PtreNRT* Genes

Tissue cultured plantlets of *P. tremula* were grown on MS medium [83] in an air-conditioned room (22°C air temperature, 30% humidity, and day/night length of 16/8 h, light intensity of 63–70 µmol m^−2^ s^−1^) for three weeks. Afterwards, the plantlets were transferred to Long-Ashton hydroponic solution (0.02 mM KNO_3_, 0.90 mM Ca(NO_3_)2•4H_2_O, 0.30 mM MgSO_4_•7H_2_O, 0.60 mM KH_2_PO_4_, 0.04 mM K_2_HPO_4_, 0.01 mM H_3_BO_3_, 0.002 mM MnSO_4_•H_2_O, 0.007 mM Na_2_MoO_4_•2H_2_O, 0.00001 mM CoSO_4_•7H_2_O, 0.0002 mM ZnSO_4_•7H_2_O, 0.0002 mM CuSO_4_•H_2_O, EDTA-Fe, pH 5.5) and grown in the same room for three weeks. Then the *P. tremula* plants were transferred to compost soil (Kompostwerk GmbH, Niederorla, Germany) and kept in a greenhouse (22°C air temperature, 30% humidity, day/night length of 16/8 h, light intensity of 70–100 µmol m^−2^ s^−1^) for eight weeks from July to September.

For RNA extraction, the plants were harvested. The top (2 cm upper stem segment (T)) was frozen after removal of young leaves. The remaining stem was separated into bark (B), developing xylem (DX), and wood (W). The developing xylem was collected by scraping the exposed surface of wood. Leaf discs (L) were cut out from the fully expanded leaves with a cork borer (diameter 1 cm). Petioles (P) were separated from the leaves. Roots were divided into fine roots (FR) and coarse roots (CR). Each of the harvested tissues from 20 plants were pooled and split to two replicates for RNA extraction. All harvested samples were shock frozen in liquid nitrogen and stored at –80°C.

### Identification and Phylogenetic Analysis of NRTs in Poplar

The sequences of NRTs in *Arabidopsis thaliana* were obtained from TAIR (http://www.arabidopsis.org/index.jsp). *Arabidopsis* 58 NRTs [12] were used to search against the proteome database in HMMER (http://hmmer.janelia.org/). Nitrate transporter was used as key word to search against poplar genome database (http://www.phytozome.net/) and National Center for Biotechnology Information (NCBI) gene databases (http://www.ncbi.nlm.nih.gov/). After removing the redundant, the remaining sequences were further used for the phylogenetic analysis.

Multiple amino acid alignments of NRTs were generated using Clustal X2 (http://www.clustal.org/) (Larkin et al. 2007). The phylogenetic trees were constructed using the neighbor-joining method with the bootstrapped option n = 1000 in MEGA 5 (http://www.megasoftware.net/) [84]. Based on the results of the phylogenetic analysis, the *P. trichocarpa* NRT1s were named as PtNRT1+clade name.

Trans-membrane domains were predicted by TMHMM v2. (http://www.cbs.dtu.dk/services/TMHMM/).

### *Cis*-regulatory Elements of *NRT* Genes in Poplar and *Arabidopsis*

The 1 kb upstream of all *NRT* genes in *Arabidopsis thaliana* and *P. trichocarpa* were obtained from ENSEMBL plant (http://plants.ensembl.org/index.html) and phytozome (http://www.phytozome.net/), respectively. The *cis*-regulatory elements were scanned in PLACE [85] and Cistome (http://bar.utoronto.ca/cistome/cgi-bin/BAR_Cistome.cgi) with the following settings: Ze cutoff = 3.0 (0< z <10), functional depth cutoff for PSSMs = 0.35 (0.0< d <1.0), and the proportion of genes the motif should be found in = 0 to discover all the motifs in each family genes (0.0<p<1.0). Both strands were searched.

For CRE analysis only NRTs found in *P. trichocarpa* were usable. Overlapping CREs were documented by Venn diagrams [86]. To generate a tissue-CRE matrix, the total number of elements per motif was counted for the NRTs expressed in each tissue. The matrix was analyzed by nonmetric multidimensional scaling (NMDS) in the open access software PAST version 2.17c (http://folk.uio.no/ohammer/past/) using the Bray Curtis index as similarity measure [87].

### Transcript Analysis using Affymetrix Genechips

The published datasets of Affymetrix genechip analyses were downloaded from Arrayexpress (http://www.ebi.ac.uk/arrayexpress) (Table S2). All Affymetrix based CEL files were transformed to signal intensity values using the affy package from R software (http://www.bioconductor.org). Expression heatmaps were generated in R using package gplots (http://cran.r-project.org/web/packages/gplots/index.html).

### Primer Design for the Quantitative RT-PCR

Based on functionally characterized NRT genes in Arabidopsis (9 *NRT1,* 7 *NRT2* and 2 *NRT3*), their homologs in the poplar (15 *NRT1,* 6 *NRT2* and 5 *NRT3*) were used for the qRT-PCR analysis. To develop specific primers of poplar *NRT* genes multi-alignment analysis for the coding sequence was conducted by the software ClustalX2 and web-server MultiAli (http://multalin.toulouse.inra.fr/multalin/). Exon and intron information within the coding sequence was obtained from website phytozome (http://www.phytozome.net/) and Gene Structure Display Server (http://gsds.cbi.pku.edu.cn/). To improve the quality of primers and prevent missmatch and DNA contamination, the specific primers were obtained based on the condition that the selected sequences are on the non-conserved regions. Exon-exon junction primers or exon-exon primers with intron inside were developed. Finally, 21 specific primer pairs for qRT-PCR were designed with a predicted melting temperature (Tm) of 60±2°C, primer length of 18–24 nucleotides, product size of 100–250 base pairs and a GC content of 40%–60% using web server Primer3 from SimGene.com (http://simgene.com/Primer3). The details are shown in Table S4.

For eight *NRT* genes exon-exon junction primers were designed. For seven *NRT* genes exon - exon primers with an intron inside of the amplified product were designed. For six *NRT* genes only exon specific primers were found. All the primer pairs were tested in the plant leaves of *P. trichocarpa* and *P. tremula*. In order to distinguish the *NRT* transcript in two poplar species we named the *NRT* in *P. trichocarpa* as *PtNRT* and the *NRT* in *P. tremula* as *PtreNRT* in our study. Because of the high similarity and properties between the coding sequence of following gene pairs: *NRT1.5A* and *NRT1.5B* (92%); *NRT2.4A* and *NRT2.4B* (97%); and *NRT3.1B* and *NRT3.1C* (97%), the primers were designed on the common sequences of *NRT1.5A* and *NRT1.5B*, *NRT2.4 A* and *NRT2.4B*, and *NRT3.1B* and *NRT3.1C,* and named as *NRT1.5A*, *NRT2.4A*, and *NRT3.1B*, respectively.

### RNA Isolation, cDNA Synthesis and Quantitative Real Time PCR

Total RNA of the samples was extracted using the cetyltrimethyl ammonium bromide (CTAB) method [82]. The RNA integrity was checked by 2% agar gel electrophoresis. DNA contamination was removed by DNAase using the TURBO DNA-free Kit according to the manual instructions (Applied Biosystems, Darmstadt, Germany). The extracted RNA was subjected to PCR with *ACT1* (POPTR_0001s31700) primers (Table S4) to check if DNA was completely removed. The PCR amplified solution was run on a 2% agarose gel to make sure no product show on the gel. Then, first-strand cDNA synthesis was carried out with approximately 1 µg RNA using a First Strand cDNA Synthesis Kit (MBI-Fermentas, St.Leon-Rot, Germany). To evaluate the quality 0.2 µl cDNA was subjected to PCR using primer pairs of *ACT1* and run on a 2% agarose gel. The prepared cDNA was used for the qRT-PCR analysis. PCR amplification was run for 35 cycles with denaturation at 94°C for 30 seconds, annealing at 60°C for 30 seconds, and extension at 72°C for 30 seconds in a LightCycler® 480 Detection system (Roche Diagnostics, Mannheim, Germany) using SYBR Green Master kit (Roche Diagnostics, Mannheim, Germany). To calculate the relative expression of the gene in each sample, the 2^−ΔΔCT^ method was used [88].

## Supporting Information

Figure S1 **Cluster analysis of** ***cis*** **-regulatory elements of** ***PtNRT*** **genes.** Color key represents the copy numbers of CREs in 1 kb promoter regions of *PtNRT* genes. The color of the gene name represents the clade in the phylogenetic tree (Figure 1). The analysis resulted in nine clusters of *PtNRT* genes (GC1–GC9) and 14 clusters of CRE motifs (MC1–MC14).
(TIF)

Table S1 **Identification of** ***NRT1/PTR1*** **,** ***NRT2*** **, and** ***NRT3*** **genes in** ***P. trichocarpa (Populus trichocarpa v3. genome*** **).**
(XLSX)

Table S2 **Microarrays used for the transcript analysis of** ***Populus NRT*** **genes in leaves, bark, wood, developing xylem, stem top in the elongation zone, and roots.**
(XLSX)

Table S3 **Mean expression level and motif abundances of** ***PtNRT*** **family genes.**
(XLSX)

Table S4 **Specific primers for** ***NRT*** **genes and housekeeping genes in** ***Populus*** **.**
(XLSX)
