# Supplementary figures and images for: The Nitrate Transporter (NRT) Gene Family in Poplar
Source: PLoS One. 2013 Aug 19;8(8):e72126. doi: 10.1371/journal.pone.0072126 (PMC3747271; doi:10.1371/journal.pone.0072126)

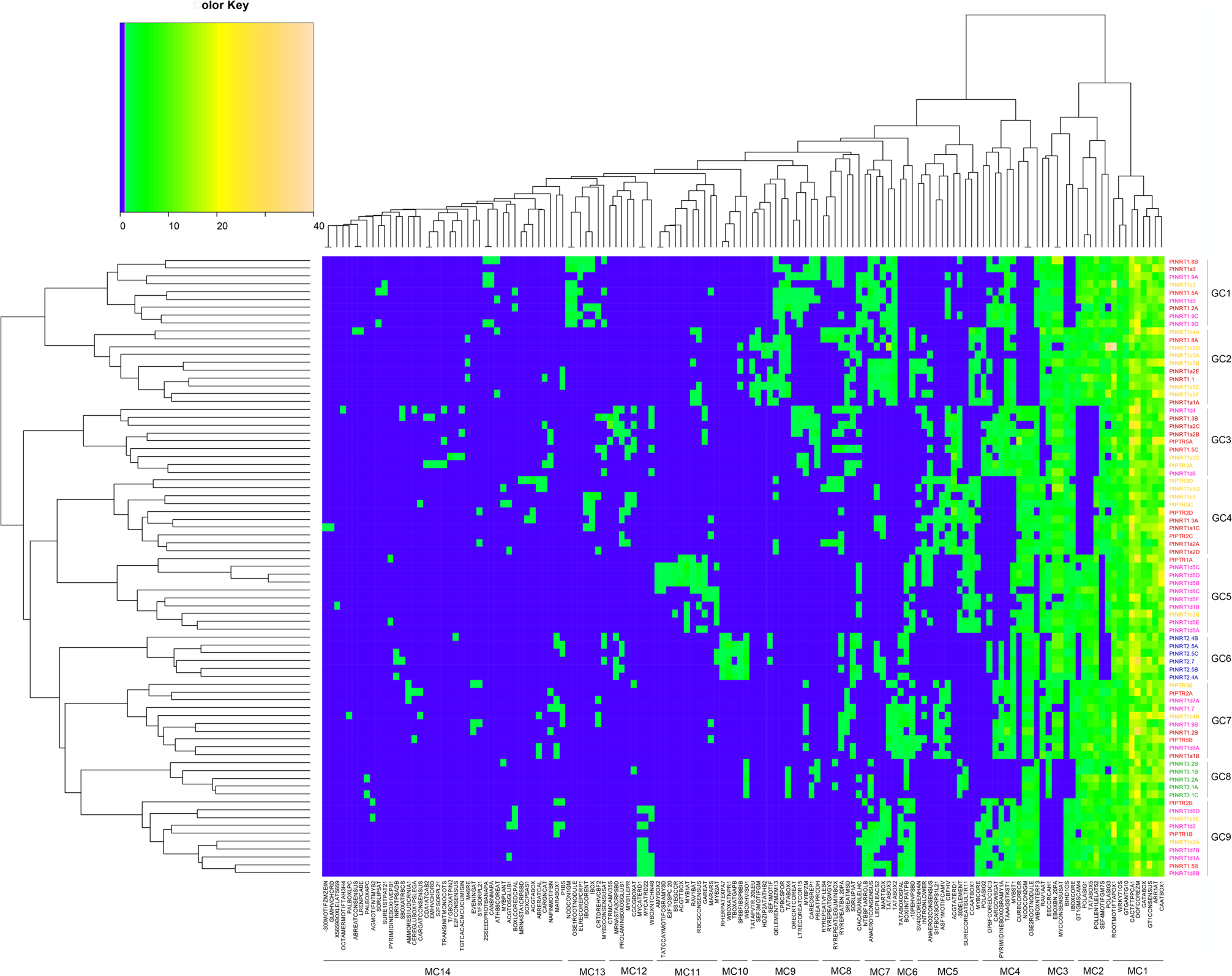

Supplement: Figure S1 — Cluster analysis of cis -regulatory elements of PtNRT genes. Color key represents the copy numbers of CREs in 1 kb promoter regions of PtNRT genes. The color of the gene name represents the clade in the phylogenetic tree (Figure 1). The analysis resulted in nine clusters of PtNRT genes (GC1–GC9) and 14 clusters of CRE motifs (MC1–MC14). (TIF) [file pone.0072126.s001.tif]
